# Role of Saliva as a Non-Invasive Diagnostic Method for Detection of COVID-19

**DOI:** 10.7759/cureus.27471

**Published:** 2022-07-29

**Authors:** Supratim Ghosh, Akshay Dhobley, Kishan K Avula, Shali Joseph, Neelam Gavali, Shradha Sinha

**Affiliations:** 1 Department of Oral and Maxillofacial Pathology, Dr. R Ahmed Dental College and Hospital, Kolkata, IND; 2 Department of Oral Pathology, Government Dental College & Hospital, Nagpur, IND; 3 Dentistry, Anusri Speciality Dental Clinic, Vishakhapatnam, IND; 4 Periodontology, Amrith Educational and Cultural Society (AECS) Maaruti College of Dental Sciences and Research Centre, Bangalore, IND; 5 Department of Periodontology, Bharati Vidyapeeth University Dental College & Research Institute, Pune, IND; 6 Department of Periodontology, Oxford Dental College, Bangalore, IND

**Keywords:** non invasive, chairside, diagnosis, covid 19, saliva

## Abstract

SARS coronavirus2 is the primary cause of new Coronavirus illness (COVID-19) (SARS- COV-2). Today, COVID-19 is a global epidemic. Coronavirus illness may be diagnosed using a variety of approaches. The gold standard is RT-PCR, which is used in all of these assays. Swabs from the nose, pharynx, or mouth are the most often used sampling methods for coronavirus detection. For COVID-19 testing, saliva may be utilized as an alternate sample. When compared to a nasopharyngeal swab, saliva samples have a number of advantages and disadvantages. Saliva has also been reviewed as a non-invasive diagnostic tool for the detection of COVID-19. The affordability of the salivary diagnostic process makes it an effective process for detecting the COVID-19 viruses. The researchers have found that salivary diagnostic processes have greater chances of success than other processes of Coronavirus detection. However, healthcare professionals need to make positive changes to their working processes to ensure the sustainability of the salivary diagnosis processes.

## Introduction and background

As of December 2019, in Wuhan, an unidentified coronavirus (SARS-CoV-2) has been isolated from a patient population suffering from pneumonia. Coronaviruses are large, non-segmented, single-stranded RNA viruses having a viral RNA genome of 27 to 32 kb. The genome consists of nucleocapsids encompassed by envelopes. Three structural proteins aid in viral entry into the host: envelope (E), membrane (M), and spike (S). The large protrusions of the spike proteins give the appearance of possessing a crown (therefore, the term corona derives from the crown) [1].

Angiotensin-converting enzyme 2 (ACE2) receptors on human alveolar sort II cells are initially linked to the spike protein of the SARS-CoV-2 virion because of their high affinity. Next, the spike protein is cleaved by the Transmembrane protease, serine 2 (TMPRSS2) protease, which then delivers viral ribonucleic acid (RNA) into infected cells. Virion release is facilitated by exocytosis, and it may infect liver cells, intestines, kidney cells, T lymphocytes, and lower respiratory tract cells [2].

There is a wide range of clinical manifestations of COVID-19. Flu-like symptoms such as a runny nose and a runny or persistent pain and sore throat may accompany other symptoms, including shortness of breath and a dry cough. Acute respiratory distress syndrome (ARDS), severe pneumonia, and multiorgan dysfunction are among the possible side effects of COVID-19 [3].

Viral culture, which takes up to seven days for the test results, and molecular approaches such as real-time reverse transcription polymerase chain reaction (rRT-PCR), isothermal amplification, and clustered regularly interspaced short palindromic repeats (CRISPR) based methods may be used to identify COVID-19. Respiratory samples are often tested for viral RNA using RT-PCR (reverse transcription) or real-time PCR (real-time polymerase chain reaction) (oropharyngeal swabs, sputum, nasopharyngeal aspirate, deep tracheal aspirate, or bronchoalveolar lavage which goes up to 2-3 cm inside the oropharynx region) [4].

Because SARS-CoV-2 is found in saliva, saliva may be used as a non-invasive diagnostic method for the identification of COVID-19 [5].

Saliva is a clear, complex fluid with a slightly acidic pH, secreted by the various major and minor salivary glands. Generally, 1-1.5 L of saliva is produced daily by the salivary glands. It consists of a variety of hormones, antibodies, enzymes, growth factors, and antimicrobial constituents [6]. In ancient traditional Chinese medicine, blood and saliva have been stated as "brothers" in the body, having a similar origin. Additionally, saliva has been utilized for over 2,000 years to diagnose diseases in humans [7].

Since saliva plays a key role in the dissemination of the virus. The severe acute respiratory syndrome (SARS-CoV-2) virus was first discovered in the Wuhan area of China during a pneumonia outbreak. The researchers first indicated that the salivary diagnosis process can be implemented to measure the detection of the SARS-CoV-2 viruses. The initial outbreak across the Wuhan area was critical as WHO was forced to declare COVID-19 as a global pandemic at the end of March 2020. The COVID-19 pandemic has caused an increase in the number of confirmed COVID-19 cases to 4,789,205. Approximately 213 countries have suffered the impact of the COVID-19 virus as the total number of confirmed cases reached 318,789 [8].

The massive number of deaths associated with the COVID-19 outbreak was primarily due to the contamination processes associated with the viruses. The researchers initially faced major difficulties, such as the unavailability of the exact information about the virus and the exact spread amongst the individuals in finding safe processes to analyze the impact of the viruses. Saliva can be seen as the host for multiple viruses associated with human bodies. The viruses gathered within saliva can cause respiratory infections in the human body.

Scientists realized that the salivary diagnosis process could be used as an effective way of determining the pathogenesis. A major advantage of the salivary diagnosis process is the presence of the Angiotensin-Converting Enzyme 2 (ACE2) within the oral cavity cells. The presence of the ACE2 receptor is responsible for allowing the SARS-CoV-2 viruses into the human body [9]. The researchers realized that collecting the saliva samples would work as an effective process for segregating the people that contracted the virus from healthy people. The process gained popularity among researchers as the saliva collection process can be implemented by creating a laboratory environment for collecting samples. This research tries to understand the several advantages researchers had due to the implementation of salivary diagnosis processes in tackling the COVID-19 pandemic. In this short review, saliva has been considered as a non-invasive diagnostic tool for the detection of COVID-19.

## Review

Saliva as a biological fluid

Both the main and minor salivary glands in the mouth cavity produce saliva, which is a distinct extrinsic fluid.

Among the principal constituents of the saliva, water predominates (almost 94%-95% of the volume). The other ingredients include inorganic molecules like potassium, sodium, calcium, chloride, magnesium, bicarbonate, phosphate (0.2% approx.), and the organic molecules that account for approximately 0.5%. All the ingredients are associated with the different functions of saliva, including digestion of food, lubrication of oral mucosa, protection, and cleaning of the oral cavity [10,11].

Salivary glands, oral mucosa transudate, gingival fold, and mucus released from the nasal cavity and throat are all components of the whole saliva. This sample also has bacteria that don't stick to the mouth, chemicals, food particles, dead epithelial cells, and blood cells [11].

Salivary flow

The average person produces 1 to 1.5 liters of saliva per day. Based on the salivary flow (SF) Index, the salivary flow may be classified as normal, low, or extremely low [10].

Effect of saliva on microorganisms

Saliva provides an ideal environment for the growth and colonization of various oral microorganisms within threshold limits, thereby maintaining the hemostasis of the oral cavity. In the salivary pellicle, proteins such as acid proline-rich protein, statherin, and histatins enhance oral bacterial settlement on tooth surfaces. Moreover, they are the source of nutrition for the oral bacteria, thereby helping them to survive and reproduce. Saliva contains mucin and glycosylated proteins that act as the source of growth and metabolism of oral microbes by providing carbon and nitrogen. Saliva plays a vital role in preventing pathogens from entering the respiratory and gastrointestinal tracts, thus serving the purpose of a "gate keeper" [12].

Various immune responses

Besides all this, saliva contains various antimicrobial and antiviral proteins that play a vital role in the innate immune response. The antimicrobial components include lactoferrin, lysozyme, peroxidase, and histatins. The secretory IgA present in the saliva prevents the adhesion and colonization of pathogenic microorganisms by binding to its surface molecule. Antiviral activities are also seen among most of the antimicrobial components like cathelicidin (which involves the disintegration [damaging and puncturing] of cell membranes of organisms), lysozyme, peroxidase, mucin, lactoferrin, & defensins, and salivary immunoglobulin A (sIgA), salivary agglutinin, and cystatins. They directly bind to the virus, thereby inactivating it, or indirectly by modulating the signal pathway, intracellular modulation of viral replication, and recruitment of immune cells inside it [10]. The salivary microvesicles, along with micro RNA, exhibit antiviral activity by inhibiting the replication of some types of viruses [9]. This microRNA can imitate various molecular events in the cell, thereby making them an associated marker to identify the infection status in the cell. It has diagnostic importance in cases of low viral replicative conditions, where the virus is not present in the saliva. Moreover, it also helps in assessing the pathological effect of the virus associated with disease [5].

So, there is a distinct correlation between the reduction in saliva flow and an increase in the severity of respiratory infections. Various analyses performed to relate the behavior of the virus with the composition and biological function of the saliva indicate that virus transmission is associated with saliva [12]. 

Saliva is an important biological fluid present within our system that helps in digesting our foods. Saliva is comprised of components such as electrolytes, mucus, immune cells, and epithelial and protective proteins [13]. The presence of these components helps the researchers to implement testing procedures with the help of saliva samples. The percentage of water associated with human saliva is close to 95%. Both organic and inorganic cells are a part of the saliva. Approximately 0.5% and 0.2% are the numbers of organic and inorganic cells present within saliva. The presence of microorganisms within the saliva provides a clear indication of the colonization processes associated with a human. Researchers can use the microorganisms found in saliva to detect the presence of dangerous viruses within human bodies that can cause fatal complications.

The SARS-CoV-2 Virus Has the Following Three Processes Present in Saliva:

The lower and upper respiratory tracts can be used as hosts for the SARS-CoV-2 viruses inside the human body. The virus can reach the oral cavity of humans through liquid droplets.

SARS-CoV-2 can use the gingival crevicular fluid as a fluid to enter the human system [14]. The fluid present in the saliva can assist the virus to enter the human body through the mouth.

The infections present within the salivary glands can cause the contraction of the COVID-19 virus, as both major and minor infections are helpful for the virus to enter the human body. The release of particles with the help of salivary ducts is enough for the virus to enter the human system.

Detection of the COVID-19 pandemic with the help of saliva provides a 90% success rate for researchers and doctors [15]. The highest percentage of success rate is a primary reason why the salivary diagnosis process has gained massive popularity among researchers in detecting COVID-19 virus. The simplicity associated with the salivary diagnosis process is another major reason why researchers have adopted this diagnosis process for detecting the COVID-19 virus. 

An overview of COVID-19

There are 26 species of coronavirus in the order Nidovirales, which may be split into four genera. The family Coronaviridae includes the coronaviruses. And its genus is the two most common human pathogens. The coronaviruses like SARS-CoV, SARS-Cov-2, and MERS-CoV all belong to the genus [12].

The structure of coronavirus: RNA virus has a diameter of 50-200 nm. SARS-CoV-2 is a positive-sense and single-stranded RNA virus. Structure proteins include S, E, M, and N. The S (Spike) protein is responsible for viral envelope generation and facilitates attachment, fusion, entry, and transmission into the host cell. The N (Nucleocapsid) protein carries the RNA genome. The M (membrane) protein is responsible for viral envelope generation. The E (Envelope) protein is responsible for viral envelope generation [5].

SARS-CoV-2 genes have numerous sequences, such as E, RdRp, N1, N2, and S, that act as the targets for RT-PCR-based tests. Enzyme immunoassays use SARS-CoV-2 antigens and immunoglobulin produced against them for detection [5].

Several clinical studies revealed that SARS-Cov-2 infection frequently presented with a wide range of symptoms, including common flu-like symptoms such as fatigue, headache, fever, dry cough, and shortness of breath, as well as anosmia, ageusia, and gastrointestinal symptoms such as nausea, vomiting, and diarrhea. Many cases often remain asymptomatic, which can act as a silent source of disease transmission to healthy subjects, and hence can be termed as "super spreaders." SARS-incubation Cov-2's period ranges from one to 14 days, with an average of three to seven. The SARS-basic CoV-2's reproductive number (R0) says that one infected person can spread the virus to between 1.4% and 3.9% of healthy people [16].

When it comes to the angiotensin II receptor, SARS-CoV2 is particularly fond. The S protein binds with the ACE-2 receptor by activating host type II transmembrane serine protease TMPRS2 on the primary target cell [5] to gain entry into the host cell. The ACE-2 receptor was most highly expressed in tongue epithelial cells, followed by buccal and gingival tissue [12] and oral fibroblasts in oral tissues [5]. Minor salivary glands also express the ACE2 receptor, which means they could be a possible target for COVID-19 [11]. The early SARS-Cov-2 infection may have been caused by the virus that is released from the salivary glands [12]. Thus, saliva plays a vital role in the detection as well as transmission of SARS-Cov-2, especially in asymptomatic cases [11].

Different studies show that the amount of SARS-CoV2 virus in saliva is higher than in a throat swab when tested with Real-Time RT-PCR. The greatest detection rate is reported after four days of illness development and during the advancement of the infection into the lungs. To find SARS-Cov-2, it is crucial to know where and when to collect specimens from different places and times [16].

The COVID-19 pandemic is creating major difficulties for world health organizations as the virus is constantly changing its structural properties. The mutation process associated with the COVID-19 virus is creating major difficulties for doctors and researchers in finding the necessary cure for the disease. The SARS-CoV-2 virus has RNA polymers that are tightly packed into the center of the particle. The center of the particle is surrounded by the protective capsid [17]. The protective capsid surrounding the center particle is comprised of a lattice of repeated proteins. Capsid proteins are another name for the lattice.

Coronavirus has a nucleocapsid and an RNA viral genome which are responsible for the creation of the inner part of the virus [18]. The envelope works as a protector for the RNA viral genome that reacts with the inner cells of the organs after entering the human system.

There are multiple signs and symbols associated with the coronavirus. According to the researchers, patients can detect the presence of the virus through primary symptoms such as fatigue, common cold, and fever. The continuous fever among the contracted patients can be used as a primary symbol for the contraction of the virus. Other symptoms, such as a runny nose, fever, and loss of smell, can be used to determine whether or not a person has contracted the virus [19].

Immune hyperactivity is a major implication of the COVID-19 virus. Researchers have conducted several studies that show that patients who contracted the COVID-19 virus are facing difficulties with their recoveries if they have pre-existing immunity-related issues with their system. Viral replication and pulmonary destruction are known as other pathogenic problems associated with the COVID-19 virus. The contraction of the virus is creating difficulties for patients with pre-existing diseases. The infection associated with the COVID-19 virus is causing major difficulties for doctors in containing the spread of cellular infections. The SARS-CoV infection combined with the MERS-CoV virus is causing major difficulties in managing problems associated with vascular congestion and systematic inflammation [20]. The process of pathogenesis associated with the COVID-19 virus is causing major difficulties for researchers in prescribing solutions for patients. The mutant nature of the virus is causing difficulties in finding care for patients that have pre-existing issues associated with their bodies.

Studies have indicated that researchers are finding it difficult to contain the effects of the COVID-19 pandemic after the contamination of the virus. Precautions need to be considered while tackling the virus as the health and safety of the surrounding people may be compromised [21]. The health officials are told to be tested with the help of RT-PCR tests regularly to understand their level of safety. 

The importance of saliva for the diagnosis of COVID-19

There is an ACE-2 receptor in the salivary epithelium, which when infected with SARS-Cov-2, may induce acute sialadenitis symptoms such as pain, irritation, and inflammation in the primary salivary glands. An increase in the concentration of salivary amylase in peripheral circulation is caused by SARS-CoV-2, which causes salivary glands to produce amylase. At its severe stage, it can induce chronic sialadenitis in the salivary gland [11]. 

The size of the saliva droplet has a significant impact on transmission. Because they fly further and settle faster, large droplets (diameter > 60 m) reduce the danger of transmission to others in close proximity to the suspect. The culprit's microscopic droplets (diameter 60 m) may spread among anyone within a one-meter radius around the suspect. But it is capable of long-range transmission if the size drops below 10 m (aerosols). The salivary droplets are also produced during coughing, speaking, and sneezing, but the size, distance, and quantity vary from person to person. Each cough can produce 3000 salivary droplet nuclei that increase to 40.000 droplets during sneezing. During breathing, on exhalation, the droplet can exceed one meter in the air [11].

Route of entry into saliva: SARS-CoV-2 uses three separate routes of entry into saliva. The lower and upper respiratory tract, liquid droplets, blood, gingival crevicular fluid, and salivary gland are the routes mentioned [11].

Salivary ducts: Saliva has diagnostic value in COVID-19 because reverse transcription-quantitative polymerase chain reaction (RT-qPCR) is the gold standard method for detecting SARS-CoV-2 (RT-qPCR) [11]. A positive result in RT-PCR confirms the presence of the virus in the sample. But a negative result can be false due to an error in timing and poor handling of the samples. The low viral count and even mutation of the viral genome also account for false-negative results [13]. So, there is a need for a chest radiograph or chest computed tomography (CT), which will show the presence of bilateral pneumonia in combination with multilobular ground glass opacities asymmetrically distributed in the posterior and peripheral regions of the lung [11].

On March 19^th^, 2020, the World Health Organization (WHO) recommended that RT-PCR should be performed to identify SARS-Cov-2 using samples from the upper and lower respiratory tracts. In order to identify early viral infection, swabs from the nasopharynx and oropharynx are used as upper respiratory specimens. Samples of bronchoalveolar lavage and endotracheal aspirate are examples of lower respiratory specimens, but they put health professionals at risk of aerosol/droplet transmission [16]. 

Various data suggest that nasopharyngeal swabs have a greater chance of giving positive results (53.36%-73.3%) in comparison to oropharyngeal swabs, irrespective of the severity of the disease condition. The sputum specimen also displayed higher positive results [22]. Although these are the commonly used procedures to detect SARS-CoV-2, they have various drawbacks. The acceptability of the patient is more important since many patients need to go for multiple numbers of testing procedures during the disease. In all the above techniques, the patient acceptance level is not up to the mark due to the discomfort during collection, and the chances of bleeding during the procedure are also present. To prevent cross-infection, healthcare professionals must also take various precautionary measures, such as using PPE kits during specimen collection. In addition to that, they need special training to collect the sample and preserve it, along with the expensive kits used for testing [11].

Saliva plays a critical role in providing an effective opportunity for researchers to test the COVID-19 virus. The following are the prime reasons why saliva has gained a massive advantage in testing the contamination of the COVID-19 viruses.

Procedural simplicity

The saliva detection process can be conducted by following simple steps by healthcare professionals. The rules and regulations associated with the testing process are quite simple, and officials can carry out the process across a variety of locations. Healthcare professionals need to follow a set of rules and regulations for conducting the tests associated with the COVID-19 virus [23]. The RT-PCR (Real-Time Reverse Transcription Polymerase Chain) testing process has emerged as an effective way of using saliva to detect the presence of the COVID-19 virus within the human body.

Accurate analysis

The detection of coronavirus using saliva provides an effective process for the researchers to find accurate test results for patients. Saliva is comprised of micro-organisms that help researchers detect the presence of SARS-CoV-2 viruses within the human body [24]. The high accuracy level of the RT-PCR tests has encouraged researchers to use the salivary detection process to find the contamination of the COVID-19 viruses.

The point of entry

The coronavirus has several points of entry into the human system. The respiratory tract, blood, salivary glands, and liquid droplets can be the entry points associated with coronaviruses. The salivary design is a process that enables researchers to use minimal resources to find the contamination of viruses in the human body. Salivary diagnosis can be used to determine the effects of the coronavirus at entry points across the nose and throat. The salivary diagnosis process helps the researchers to implement tests without creating major difficulties or complexities associated with the testing procedures.

Patient comfort: Patient comfort is important when diagnosing and treating the COVID-19 virus. The salivary diagnosis process can be completed without creating major difficulties for patients. Doctors and healthcare professionals can determine the test results within a very limited amount of time [25]. The doctors can conduct the salivary diagnosis process multiple times, and the patients will not face major complications in completing the RT-PCR tests. 

So, the researchers must search for procedures that do not hamper patient comfort and are cost-effective as well. As a result, a new study shows that human saliva may be a viable option for the detection of SARS-CoV-2 infection shortly. To gather, there is no requirement for an expert in the field [22].

Advantages of salivary samples: Saliva can be used as a diagnostic fluid for several reasons, such as minimally invasive procedures, being inexpensive, and easy handling and storage; it has a real-time diagnostic value; a large portion of patient comfort is independent of age (from children to elderly patients) [5]. Previous studies revealed that the viral load in the saliva is around 108 to 109 particles/mL, so it has a high amount of viral load in it. Saliva is made up of secretions from the respiratory tract and oropharyngeal mucous epithelial cells. This means that with one sample, we have many targets, which makes it more likely that a virus will be found [22].

The following are multiple advantages associated with the salivary diagnosis process:

Time consumption: The time consumption for conducting the salivary diagnosis process is minimal. Healthcare professionals can conduct multiple tests within a single day using the salivary diagnosis process. Healthcare professionals need to follow a few precautionary steps, such as proper masking, using new toolkits, and sanitization for conducting the salivary diagnosis process [26]. The safety steps don't take a lot of time between each sample collection, which makes it a good way to get saliva samples from patients.

Differentiating between symptomatic and asymptomatic patients: The salivary diagnosis process provides an effective opportunity for healthcare professionals to differentiate between symptomatic and asymptomatic patients by the parameters such as viral load and virus detection. The saliva samples collected from patients can be evaluated in an effective way to differentiate between patients [27]. The preventive method may be implemented since the salivary diagnostic procedure is inexpensive.

Restricting the chain of the virus: The salivary diagnosis process can be implemented to create a bio-bubble. The bio-bubble process has been used as an effective process for restricting the flow of COVID-19 viruses. The bio-bubble process is comprised of the salivary diagnosis process as people who are incorporated within the bio-bubble are constantly tested to determine their physical condition. The quick test results provide an effective advantage for the event organizers to protect against the COVID-19 viruses.

The bio-bubble process is used for conducting major sporting events around the world. Various tests which are available for rapid diagnosis are:

The molecular test sometimes referred to as the RT-PCR test, is a type of testing used to identify viruses in samples directly. The test looks for the virus's RNA, which is also its genetic material. The reverse transcriptase enzyme is used in the first phase of this test to turn this RNA into DNA. This DNA is then found via PCR analysis. RT-PCR was thus named. These are also known as the nucleic acid amplification test and the diagnostic test (NAAT). A throat or nasal swab sample is obtained. Results are either immediately or within a couple of days. The best tests are those mentioned above. They are extremely sensitive and particular. These examinations aid in the coronavirus infection's active diagnosis. A positive test indicates that the virus was present in the sample, but a negative test does not rule out the possibility that you were infected at the time you took the test. It doesn't indicate whether you've ever had COVID-19.

The COVID-19 antigen test aids in locating the COVID-19-related antigens. A rapid diagnostic test called an antigen test, sometimes referred to as a rapid antigen test, provides findings more quickly than molecular testing. However, antigen testing has the disadvantage of being more likely to overlook an infection that is already active. Typically, a throat or nasal swab sample is obtained. Results are provided within an hour or less because it is a quick test. If you test positive, rapid tests are often quite reliable; however, a negative result might need to be verified by a molecular test. These examinations aid in the coronavirus diagnosis in the sample. Compared to RT-PCR assays, the antigen test may fail to detect a current coronavirus infection.

Screening tests for COVID-19 antibodies in your blood are known as serology testing, serological tests, or serology blood tests. It reveals whether you have ever had the virus that causes COVID-19. The antibody test examines your immune system's response to the infection rather than looking for the actual virus. By taking a sample of your blood, it is examined. Results are supplied either immediately or within one to three days. For more precise findings, it may occasionally be necessary to do a second COVID-19 antibody test. If you have antibodies against the virus that causes COVID-19, it may be determined by this test if you have ever been exposed to it. A COVID-19 antibody test might not be able to detect the presence of an active COVID-19 virus in your body.

The quick salivary diagnosis process can be used to determine the COVID-19 contamination rates as the contacted purple can be quarantined to protect others within the bio-bubble from virus contamination. Major sporting events such as the English Premier League, national basketball league (NBA), and Euro 2020 were held within a bio-bubble where players' physical health was tested regularly [28]. The salivary diagnosis process was the first choice of the healthcare professionals to test the physical condition of the athletes, as the time consumption associated with the process was minimal.

Affordability: The salivary diagnosis process provides a financially viable option for patients to evaluate their physical condition. The financial resources required for the patients are minimal for the salivary diagnostic process. The government is providing free services such as food, antigen testing for free, free medical teleservices, free medicines, and transport services for patients that have been contacted with the COVID-19 virus. Private organizations can provide services for patients with minimal charges for analyzing the condition of the virus within their system.

Saliva collection can be done in three different ways: as a collection of the saliva from the individual gland duct (time-consuming and needs special apparatus such as Lashley cup or a modified Carlson Crittenden device), by spitting/coughing out of saliva, or by the swabbing of saliva by the sponge-like device (giving the pure form of saliva, but not used regularly) [5].

Sample collection and detection methods: The patient should only use water to rinse their mouths and not any disinfecting mouthwashes. Samples should be taken as soon as possible after four days of infection to ensure that the virus count is at its highest point [22]. It has also been identified that the saliva samples can be stored for more than two years without adding any RNA inhibitors in Trizol at -80 °C. Saliva RNA was extracted using the PerkinElmer Chemagic 360 and the Chemagic Viral DNA/RNA 300 Kit H96 from the Chemagic company. Various newly improved testing kits have been introduced to use saliva as a testing sample that detects viral RNA and is under development by Rutgers Clinical Genomics Laboratory. The TaqPath Combo Kit from Applied Biosystems may be used with the QuantStudio from Thermo Fisher Applied Biosystems. In different clinical trials, it has been shown that saliva samples from SARS-Cov-2 patients can be a good way to find out if they have a virus [16].

Saliva as a diagnostic tool: Different tests which have been based on saliva identified by ICMR under investigation, approved, or under trial are shown in Table 1.

**Table 1:** ICMR issued salivary tests available

S. No	Name of the Manufacturer/Developer	Name of Kit	India/ Other countries	Name of the supplier	Validation Status
1.	Real-Time Analyzers Inc.,USA	CoviSaliva Antigen Test kit	USA	Lisaline Lifescience Technologies Pvt. Ltd., Thane	Validated, Not Approved
2.	Bhat Bio-Tech India (P) Ltd.	COVID-19 Antigen Rapid Card Test (ORAL SALIVA)	India	Invex Health, Mumbai India	Validated, Not Approved
3.	Sensing Self Pte. Ltd.	S1 Covid-19 Rapid Antigen Test Kit	Singapore	Sensing Self Pte. Ltd.	Validated, Not Approved
4.	Meril Diagnostics	MERISCREEN COVID- 19 Ag Test Kit-V.1	India	Meril Diagnostics	Validated, Not Approved
5.	Capital Health Services India Private Limited	LFA for antigen detection test - deep throat saliva specimen	India	Capital Health Services India Private Limited	Validated, Not Approved
6.	Trivitron Healthcare Pvt. Ltd., Chennai (TN), India	BIOCARD Pro COVID- 19 Antigen Saliva Kit	India	Trivitron Healthcare Pvt. Ltd.	Validated, Not Approved
7.	Meril Diagnostics	MERISCREEN COVID- 19 Ag Test Kit-V.2	India	Meril Diagnostics	Under validation
8.	PCL Inc., South Korea	PCL Antigen Saliva Test Kit	S. Korea	PCL Inc.	Under validation
9.	Mediclone BioTech Pvt. Ltd., Chennai.	@sight COVID-19 Antigen Test Kit - Saliva.	India	Mediclone BioTech Pvt. Ltd., Chennai.	Kits not submitted yet
10.	ReaGene Biosciences, Bengaluru	Saliva based COVID19 Ag test kit	India	ReaGene Biosciences, Bengaluru	Kits not submitted yet
11.	S D Biosensor Inc.	Standard Q COVID-19 antigen saliva test	S Korea	S D Biosensor	Kits not submitted yet
12.	MyLab Discovery Solutions	Pathocatch COVID-19 Ag LFT device from Saliva sample	India	MyLab Discovery Solutions	Kits not submitted yet

The test using saliva work on the principle of detection of viral load in the first few days, as saliva shows higher levels during the first few days and few use real-time polymerase chain reaction; some use antigen detection to evaluate the presence of the COVID-19 virus [15].

Because saliva is thought to be a strong reservoir for viruses that are spread by oral shedding, as well as secretions from the lower respiratory tract, nasopharynx, and possibly infected salivary glands, salivary tests offer a distinct advantage over other types of testing. With >90% concordance between saliva and nasopharyngeal swabs, saliva-based diagnostic assays have also shown to have a high sensitivity and specificity for a number of respiratory viruses, including coronavirus. Saliva is a suitable specimen type for serial viral load monitoring since it is non-invasive. After the first week of symptom onset, the SARS-CoV-2 load in saliva is said to be at its peak before gradually declining. This emphasises the fact that saliva is a strong candidate for SARS-CoV-2 detection in the early stages of the illness. Saliva may be a suitable tool for disease monitoring since the temporal profile of SARS-CoV-2 load in saliva has been observed to be more consistent as compared to that of nasopharyngeal swabs. Additionally, saliva can be utilised in clinical studies to track how well antivirals are working [16]. However, because saliva might potentially spread viruses, normal procedures must be followed when collecting it and managing it thereafter. A saliva-based SARS-CoV-2 RNA detection test has already received approval through the U.S. Food and Drug Administration emergency use authorization in light of these encouraging results.

Limitations of saliva as a diagnostic sample

Despite the various advantages of the use of saliva in SARS-Cov-2 detection, it has many disadvantages too. The content and volume of saliva vary among individuals, and different medications also influence the salivary composition and quality. Last but not least, is that there is a lack of uniformity among the levels of various biomarkers in the saliva. Moreover, the presence of proteolytic enzymes such as amylase, proteases in the saliva can degrade salivary protein, thereby hampering its predictability [5].

The Following Are Major Drawbacks Associated With the Salivary Diagnosis Process:

Mutation of the virus: Mutation of the COVID-19 virus is an effective disadvantage of the salivary diagnosis process. The virus's mutation positively alters the structure of viruses, making it challenging for medical practitioners to diagnose using the salivary diagnostic method [29]. The previously mentioned saliva collection process might not be enough for the doctors to restrict the flow of the COVID-19 viruses, causing child complexity: The salivary diagnosis process can become a problematic process for handling children's related issues. The complexity associated with infants' saliva collection, such as lack of dexterity and fewer cooperation processes, is a major drawback of the salivary diagnosis process, and it may become an unhygienic process as the contamination of the COVID-19 virus can be restricted with proper precautionary actions. The saliva collection process needs to be conducted with extreme care as the process of collecting samples for patients can cause virus contamination issues. Both healthcare and testing professionals need to take maximum care in handling the salivary samples as the patient's samples can cause damage in decreasing the contamination of the viruses. 

## Conclusions

Saliva is an important biological fluid, and it also has several biomarkers that have diagnostic value. As an alternate approach for the detection of COVID-19, only a few researchers have focused on the standardisation of salivary samples for that purpose. If an additional study is done on saliva's role in COVID-19, it might provide a promising signature for COVID-19 detection.

It can be concluded that this review has been developed to provide an overview associated with the analysis of the salivary diagnosis processes. Saliva plays a massive role in the detection of the SARS-CoV-2 virus. The SARS-CoV-2 virus and other forms of coronaviruses can be detected using the salivary detection process due to its multiple advantages. A detailed analysis of the COVID-19 virus structure has been carried out in this report. The nucleocapsid and RNA viral genome are responsible for the creation of the COVID-19 viruses. The particles of the virus are structured with spikes, a membrane, and an envelope. Fevers and loss of smell have been discussed as common symptoms of the COVID-19 viruses. Several advantages, such as less time consumption and affordability, have been mentioned in this assignment. The salivary diagnosis process provides an effective advantage for researchers to differentiate between symptomatic and asymptomatic patients. The materials present within the saliva provide an effective advantage for healthcare professionals to make informed decisions regarding the contamination of the COVID-19 viruses. The mutation of the COVID-19 virus is creating major issues for researchers as the salivary diagnosis process might not be enough to understand the true analysis of the COVID-19 virus.
